# Coding culture: challenges and recommendations for comparative cultural databases

**DOI:** 10.1017/ehs.2020.30

**Published:** 2020-06-01

**Authors:** Edward Slingerland, Quentin D. Atkinson, Carol R. Ember, Oliver Sheehan, Michael Muthukrishna, Joseph Bulbulia, Russell D. Gray

**Affiliations:** 1 Department of Asian Studies, University of British Columbia, Vancouver, Canada; 2 School of Psychology, University of Auckland, Auckland, New Zealand; 3 Human Relations Area Files, Yale University, New Haven, USA; 4 Department of Psychological and Behavioural Science, London School of Economics, London, UK; 5 School of Humanities, University of Auckland, Auckland, New Zealand; 6 Max Planck Institute for the Science of Human History, Jena, Germany

**Keywords:** Cultural evolution, big data, cross-cultural databases, historical databases

## Abstract

Considerable progress in explaining cultural evolutionary dynamics has been made by applying rigorous models from the natural sciences to historical and ethnographic information collected and accessed using novel digital platforms. Initial results have clarified several long-standing debates in cultural evolutionary studies, such as population origins, the role of religion in the evolution of complex societies and the factors that shape global patterns of language diversity. However, future progress requires recognition of the unique challenges posed by cultural data. To address these challenges, standards for data collection, organisation and analysis must be improved and widely adopted. Here, we describe some major challenges to progress in the construction of large comparative databases of cultural history, including recognising the critical role of theory, selecting appropriate units of analysis, data gathering and sampling strategies, winning expert buy-in, achieving reliability and reproducibility in coding, and ensuring interoperability and sustainability of the resulting databases. We conclude by proposing a set of practical guidelines to meet these challenges.

## Graphical abstract

**Figure:**
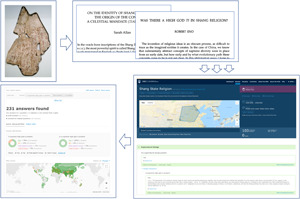

**Media summary:** This paper describes major challenges in, and proposes best practices for, the construction of large coded databases of cultural history.

Projects such as eHRAF World Cultures (HRAF, n.d.), the Ethnographic Atlas (Murdock, 1967) and the Standard Cross-Cultural Sample (SCCS) (Murdock & White, 1969) have demonstrated that the systematic study of cultural evolution on a global scale is both feasible and promising. Application of rigorous quantitative methods to these datasets has yielded novel insights into questions such as the prevalence of warfare or ritual scarification, religious ideation, global patterns of post-marital residence and descent, and links between agricultural intensification and changes in gendered work (Allan, 2007; Atkinson & Whitehouse, 2011; Ember & Ember, 1992; Guyer et al., 1988; Johnson & P, 2005; Jordan et al., 2009; Sosis & Bressler, 2003; Watts et al., 2016). For instance, eHRAF is the digital version of the Human Relations Area Files’ collection of ethnography in paper and microfiche, a collection of ethnographic data made more accessible to researchers by having individual paragraphs subject-indexed with standardised topics that characterise their content. Taking advantage of this pre-coding to guide their reading, Sosis et al. had a team of anthropology graduate students go through accounts of male rites of passage across a representative sample of eHRAF cultures to create a metric of the costliness of ritual participation (including such practices as tattooing, scarification, piercing, circumcision and body painting; Sosis et al., 2007). This metric was then compared with other variables, coded from eHRAF or other sources, concerning frequency of internal and external warfare, levels of male cooperative food production, socialisation for cooperation, subsistence type, marriage and residence patterns, etc. The authors found that the intensity of warfare was by far the strongest predictor of costly rituals. This suggests that costly rituals enable partners to reliably signal cooperative commitment where collective action is threatened by defection, rather than – as alternative hypotheses claim – functioning to signal mate value.

In recent years, the application of statistical methods from the natural sciences to cultural data has opened new possibilities for the systematic study of cultural diversity. New databases have integrated the mostly synchronic ethnographic data from more established sources such as the SCCS or eHRAF World Cultures with ecological and linguistic data (Kirby et al., 2016) and the much larger body of global diachronic historical data (Slingerland & Sullivan, 2017). Data can also now be crowdsourced, and the development of large web-accessible datasets has fuelled the use of sophisticated computational methods to analyse this data. Such advances have created the tools required to pursue a new natural history of human cultural dynamics – one that avoids long-recognised pitfalls, such as cherry-picked data (see critique in Watts et al., 2015a, b), a failure to test the generality of findings, and the problem, originally identified by Francis Galton, of the non-independence of cultures (Galton, 1889; Mace et al., 2005; Mace and Pagel 1994; Naroll, 1961; Ross & Homer, 1976). Thus, novel cultural datasets have combined with powerful computational tools to bring remarkable new clarity to central topics in the study of humans, such as human population origins (Bouckaert et al., 2012, 2018; Gray et al., 2009), the role of ritual or religion in human cooperation, the rise of complex societies (Turchin et al., 2015; Watts et al., 2015a, Watts et al., 2016) and constraints on language diversity (Dunn et al., 2011).

Despite these advances, progress remains threatened by a lack of commonly accepted standards for coding cultural data, for organising these data into formats amenable to appropriate statistical modelling and for expert validation of the quality of data-coding decisions.

Here, we focus on a representative sample of current large-scale, online databases that offer coded cultural data, represented in Table 1. We argue that coding and collating cultural data, an essential step for the comparative science of cultures, presents a series of interconnected challenges. Drawing upon our experiences in creating and administering large-scale databases, we outline what we take to be the largest obstacles to progress. We evaluate a range of possible responses to these challenges, and provide a set of specific recommendations that takes into account the unique challenges that researchers face when pursuing a science of cultural evolutionary dynamics.

**Table 1.** Representative large-scale, online cultural coding database projects

Full name	Acronym	URL	References	Type of data	Current content (* indicates expanding annually)
eHRAF World Cultures	eHRAF	ehrafworldcultures.yale.edu	http://hraf.yale.edu/products/ (Ember, 2012)	General culture (ethnographic)	320 cultures comprising ethnographic documents subject-coded at the paragraph level to facilitate searching*
eHRAF Archaeology	eHRAF	ehrafarchaeology.yale.edu	http://hraf.yale.edu/products/ (Ember, 2012)	General culture (archaeological)	102 archaeological traditions comprising archaeological documents subject-coded at the paragraph level to facilitate searching*
Database of Religious History	DRH	religiondatabase.org	Slingerland and Sullivan (2017); Sullivan et al. (2017)	Religion	397 entries on religious groups or places from 195 experts or research assistants, with coded responses to poll questions*
Pulotu: Database of Pacific Religious Beliefs and Practices	Pulotu	pulotu.shh.mpg.de	Watts et al. (2015b)	Religion	116 Austronesian cultures coded for 62 variables on religion, history, society, and the natural environment
Seshat: Global History Databank	Seshat	seshatdatabank.info/	Turchin et al. (2012, 2015, 2018)	General culture	Coded historical political, economic and religious variables for 30 ‘natural geographic areas’ around the world*
Grambank	Grambank	https://grambank.clld.org	In preparation	Grammar	195 structural features coded for over 1900 languages*
Database of Places, language, culture and environment	D-Place	d-place.org	Kirby et al. (2016)	Culture, environment, language	Cultural, linguistic, environmental and geographic information coded for over 1400 human societies
World Atlas of Language Structures	WALS	https://wals.info	Dryer and Haspelmath (2013)	Language	A large database of structural (phonological, grammatical, lexical) properties of languages gathered from descriptive materials (such as reference grammars)
Natural History of Song Project database	NHSP	https://osf.io/jmv3q/	Mehr et al. (2018)	Music	NHS Ethnography contains 50 variables coded from eHRAF for 60 human cultures. NHS Discography contains 40 variables coded from field recordings from 86 societies

## Units of analysis and data selection: recognising the critical role of theory and research goals

The comparative science of culture must organise a wealth of cultural data. Notably, any attempt to code social, historical or linguistic data inevitably requires choices about which information to collect and which units of analysis to employ. Projects should begin with a clear sense of the general type of research questions that these data are intended to answer. Research questions might be quite focussed, such as whether rates of lexical change respond to changes in population size (Bromham et al., 2015). However, when specific hypotheses too strongly drive data type selection, the resulting databases will be of limited usefulness to the broader research community. For example, a language database that consisted of indicators for grammatical complexity could not be employed to test theories of, say, phonetic change or semantic variation. Trade-offs between precision and scope suggest the need for a balance in data collection between focus and comprehensiveness.

Projects should begin with a clear set of goals, research questions or theories that will then drive the selection of appropriate units of analysis. Data gathering and coding should be preceded by broad consultation with relevant stakeholders and iterated revisions of coding procedures and rubrics. D-PLACE, Seshat, Grambank and the DRH, for instance, were built after multiple workshops with area experts to obtain feedback on variables and coding procedures.

The most difficult architectural decision, and the one that will most systematically shape the selection and coding of data, is defining the unit of analysis. This is particularly challenging when it comes to studying cultural as opposed to genetic evolution. Despite the problems involved in drawing boundaries between species or populations, defining units of analysis is considerably easier in the study of genetic evolution. Most biological variation is conveniently captured by gene sequence data, digital units that are themselves conveniently packaged in organisms. When it comes to the study of cultural evolution, what are the units of analysis?

Current large-scale projects have adopted different responses to the challenges of defining units of analysis and units of measurement, focusing on the religious group, place or object (DRH), geographically and linguistically defined culture (Pulotu, eHRAF, D-PLACE, NHSP) or polity (Seshat). When it comes to cultural information, focusing on any unit larger than the individual necessarily involves loss of some variation; the degree to which this is acceptable depends on the specific theoretically derived hypotheses driving the project. Seshat organises the world into sampling units called Natural Geographic Areas (NGAs), such as the Middle Yellow River Valley, and then slices these NGAs temporally into individual polities, spanning 200–300 years, that were present in the NGA. This may limit the resolution and nuance of the resulting data, in that any given slice of space–time in the Seshat databank will tend to represent data concerning only one particular polity, religious group or social class, while simultaneously facilitating the perception of large-scale changes over time and space by de-cluttering the signal. Seshat allows changes for individual variable codes with a Polity, and there is room in its architecture for polities, classes or groups to be coded within the same slice of space and time, but any flexibility in this regard sacrifices the analytic advantages of relatively simple units of analysis. Databases organised around cultural groups, whether linguistically or geographically defined (e.g. the SCCS or Ethnographic Atlas), often flatten the historical record and narrow to one community or district, but gain a pay-off in terms of simplicity of coding and analysis. However, not all cultural databases limit themselves to one focus. For example, eHRAF tries to cover multiple time periods and regions to capture within-culture variation. Nonetheless, HRAF recommends limiting to one focus if the researcher is trying to test a synchronic hypothesis (Ember, 2012).

All but the smallest societies have some cultural variation, but large-scale societies present a particular challenge if one wants to capture the variation in ethnicity, religion and social class or caste. One response to the challenge of dealing with large-scale societies is to begin with theoretically focused data collection while building in flexibility by allowing the modification of units of analysis or addition of new ones. The DRH project, for instance, is organised around ‘polls’, or sets of questions about a particular unit of analysis. It initially began with a single poll based on the religious group. Both the choice of ‘group’ as the unit of analysis and the selection of specific questions to constitute were driven by the desire to test certain hypotheses about the role of particular religious beliefs and practices in inter-group competition drawn from Norenzayan et al. (2016). The Group poll was subsequently expanded to incorporate questions of interest to other research teams testing alternative hypotheses. In addition, new polls organised around different units of analysis (religious place, text and object) have been introduced or are under development, in response to concerns about the coherence of the category of religious ‘group,’ and the DRH is also now hosting specialised polls designed by outside research groups focusing on other aspects of human religiosity, such as beliefs about mystical or magical harm (Singh, 2018). The result is that any given slice of space and time in the DRH can be filled by codings indexed to multiple overlapping groups, texts, places or objects. Although each poll is a self-contained database, data between polls is linked through the overlapping temporal and geographic location, explicit links between related questions, and through metadata, such as a tree of religions and geography (Sullivan et al., 2017; The Database of Religious History, n.d.). The advantages of allowing modification of units or additional units of analysis must, however, always be weighed against the costs of weakening standardisation of coding procedures. If units of analysis can be modified, automated methods for updating previous codings must be built into the system. Any new units of analysis added must be linked to previous units through some set of standardised, unchanging coding rubrics, such as religious group, language group or geographic tags.

Grambank focuses on 195 structural features that can be compared across the languages of the world using individual languages as its unit of analysis, but this then provides a platform that can be easily expanded with additional features and data types that might be specific to certain regions and language families. Seshat is organised around 30 Natural Geographic Areas (NGAs) intended to give a comprehensive global sample of cultures, with NGAs chosen in order to sample, in any given area of the globe, regions with early, intermediate or late development of complex polities. Their codebook was driven by the desire to gather historical variables relevant to testing competing hypotheses about the dynamics of cultural evolution, particularly as they relate to this rise of large-scale cooperation, including variables related to warfare, inequality, resources, rituals, administration and economics. In Seshat, an NGA (for instance, Middle Yellow River Valley) is employed as a sampling unit to obtain broad and representative coverage, with individual polities that happen to fall within these NGAs at any given time period (e.g. Western Zhou, 1122–771 BCE) functioning as the units of analysis. The Seshat team has also published explicit discussions of how coding rubrics were constructed, what questions they are intended to answer, and how uncertainty concerning codings is to be incorporated (|Brennan et al., 2016; Peregrine et al., 2018).

Whatever units of analysis are ultimately chosen, linking data to standardised, widely shared formats maximises interoperability and helps prevent initial theoretical commitments and research goals from overly limiting the manner in which the data can be accessed and analysed. Grounding all coding decisions in space and time (using GIS standards and standard date formats) and adopting standard coding formats (e.g. the Cross Linguistic Data Format for linguistic data; Forkel et al., 2018) are essential if the data is to be usable by other research teams and merged with other types of data. Linking information about the beliefs and practices of historical religions in GIS maps, for instance, allows this data to be compared with historical climate information or variables related to demography, politics, or economics. Creating a flexible data architecture, employing open source tools and using standard identifiers (including shared tagging trees, such as standardised names of religious groups) and identifier translation rubrics also greatly facilitate interoperability. For instance, D-PLACE links ethnolinguistic groups to a language classification via glottocodes (Glottolog, 2020) and to environmental data by the spatial coordinates. On the DRH platform, different polls employ different analytic entities and labels but are unified by common tagging systems for religious groups and regions, as well as the fact that all poll answers and qualitative data are ultimately grounded in space (GIS map) and time (year range). APIs (application program interfaces, discussed below) should be employed to allow databases to speak to one another. A helpful acronym coined by Wilkinson et al., FAIR, highlights the need for databases to be Findable, Accessible, Interoperable and Reusable (Wilkinson et al., 2016).

## Converting qualitative to quantitative: choosing an appropriate data coding strategy

The comparative study of genetic evolution often involves measurements (e.g. wingspan, body mass, genetic diversity) that convert qualitative physical characteristics into quantitative data. Assuming that the units of measurement are clearly indicated and data gatherers are well trained, a high degree of intercoder agreement is to be expected.

Some types of cultural data can also be coded with a high level of certainty. Questions such as whether a community has an irrigation system, grows maize or possesses bronze weapons can be coded with high reliability and require little inference. On the other hand, many types of cultural data are more challenging to convert from qualitative to quantitative form, because researchers face both a measurement and an inference problem. This process of conversion involves interpretive acts that may require extensive scholarly knowledge or individual judgment (Ember & Ember, 2001; Ember et al., 2015; François et al., 2016). Even an example that may seem straightforward, the coding of word order, is both theory dependent and involves abstracting away from the details of spatial and temporal variability. Most Slavic languages, such as Russian or Polish, have considerable flexibility in their word order. In WALS these languages are classified as having dominant verb/object order owing to a higher frequency in texts and pragmatic neutrality (see Dryer et al., 2005).

**Figure 1.**
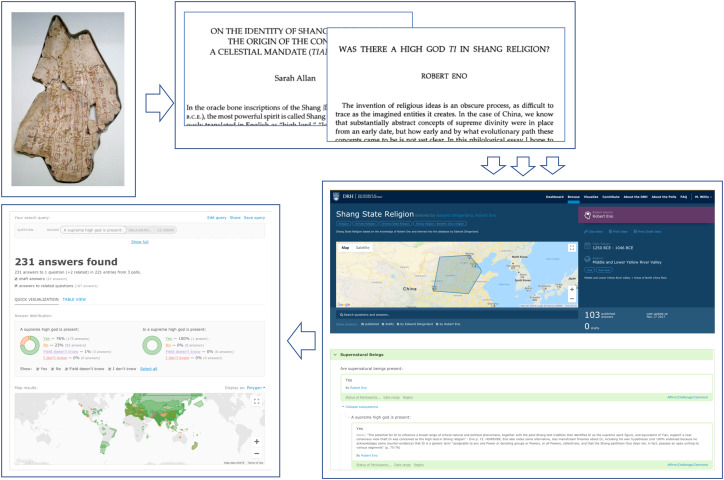
Steps involved in converting qualitative historical data to quantitative data, taken from the DRH. (1) historical texts or artifacts; (2) scholarly interpretations; (3) individual coding decisions (with justification or simply as expert opinion); and (4) quantitative data.

The challenges of coding cultural data become more apparent when it comes to areas that not only require scholarly interpretation but also involve active disagreement among experts. For instance, whether or not the Shang (c. 1600–1046 BCE) culture of China possessed a ‘high god’ remains a topic of hot scholarly debate (e.g. Allan, 2007; Eno, 1990). Figure 1 illustrates the process of coding this particular aspect of Shang culture as implemented in the DRH. The process begins with primary sources, in this case oracle bone inscriptions bearing text related to divination and other communication with supernatural beings, which are then discussed and analysed in secondary sources, such as scholarly articles in academic journals. Until the creation of large-scale, coded databases, scholars interested in analysing the dynamics of cultural evolution were stuck at stage 2, faced with the prospect of having to wade through potentially thousands of pages of qualitative discussions in the secondary literature. The DRH relies upon experts or expert-guided RAs to digest this information, in stage 3, through the process of answering a poll about the Shang Dynasty and responding categorically (Yes/No/Field Doesn't Know/I Don't Know) to questions such as, ‘A supreme high god is present’. This then yields quantitative data (step 4) that can be used to get an instant sense of opinion in the field on a given topic or to test competing hypotheses.

When it comes to converting qualitative cultural data to quantitative codings, a wide spectrum of data-gathering and data-analysing strategies is possible. At one extreme is fully automated text mining, where an algorithm is programmed to sift through a large amount of textual material in order to extract regularities, which can then be assigned codes. Once created, these algorithms require very limited person-hours to run, and can quickly process massive amounts of data. However, they are often extremely time-consuming to design, and require the data they analyse to be cleaned and formatted in particular ways, which can also be quite labour-intensive. Moreover, currently available techniques have proven to be ineffective even for tasks that might seem most amenable to such an approach, such as the extraction of typological linguistic information from published grammars (Virk et al., 2017). This may change in coming decades as artificial intelligence becomes more powerful and flexible (Virk et al., 2017), but for the moment, the need for human coders is unavoidable even when it comes to these more tractable cases.

One approach to human coding is to hire research assistants (RAs). RAs may have various degrees of academic qualification, but their distinguishing characteristic, for the purpose of this discussion, is that they do not possess expert-level knowledge of the culture or cultures being coded. The RA approach allows reliable and predictable progress in data collection, and – when RAs are properly trained – results in higher intercoder reliability than other strategies. One limitation is data bottlenecks, in that data collection can only proceed as long as funds are available to pay RAs. A more worrying limitation is that, especially when it comes to coding challenging variables covering large-scale societies, it is difficult for RAs without domain-specific training to make accurate coding decisions, considering the massive quantity of relevant primary texts and secondary literature involved. One solution, used in the Standard Cross-cultural Sample and the Ethnographic Atlas, is to narrow the focus to a community and a specific time in a large-scale society. This makes the literature more manageable for RAs.

Even more worrying, it is often impossible for underqualified RAs to even know when they do not know enough to make a coding decision or whether they might be injecting their cultural biases onto ambiguous materials. In such situations, it is especially important for non-expert coders to be given high-quality sources to consult. Another risk is that the RA coding decision will obscure a considerable difference of opinion within the scholarly community, giving a false sense of certainty. We therefore recommend that the coding of large-scale society data requiring obvious interpretive decisions (e.g. Is spirit-body dualism part of the religious belief system? Is there a high god that cares about human morality?) involves experts at some stage. The RA strategy works best when it comes to straightforward low-inference variables (e.g. Are grave goods present in burials?) or well-documented smaller-scale societies, where the quantity of relevant literature is not overwhelming.

Teams adopting the RA strategy also need to decide whether to hire large teams of coders, each of whom focuses on a small portion of data or specific case-example with which they can thoroughly familiarise themselves, or a small number of coders, each of whom codes a large amount of data. There are pros and cons to each approach. The former, by narrowing the set of variables to be coded, maximises the likelihood that two or more coders will arrive at a shared understanding of the coding scheme and is therefore likely to increase inter-coder reliability. If different coding teams are separately tasked with independent or dependent variables, the chance of bias towards the tested hypotheses are also minimised. The latter approach makes it more likely that a coder will have a more holistic understanding of difference aspects (economic, political, religious) of a given society, and this may in turn increase their ability to code higher-inference variables. On the other hand, the volume of variables coded may decrease shared understanding of the coding scheme and may result in lower inter-coder reliability for any particular variable. For either strategy, the most important ingredient for success is a well-designed, simple coding scheme. This may involve converting high-inference variables (e.g. Is there belief in an immaterial soul?) to more explicit variables (e.g. Are there mortuary practices?) and explaining the circumstances of when it is or is not appropriate to make inferences, including inferring the absence of a trait (Ember & Ember, 2009).

Turning to actual implementations, worldwide cross-cultural studies using the eHRAF databases and Pulotu, a database of religious beliefs and practices in the Pacific, can serve as examples of the two RA strategies. Using eHRAF databases, the typical approach involves: (a) pretesting the coding protocol for each variable to ensure that the coding rules are not only understood by one or more RAs, but also that there is sufficient material in the ethnographic record to be able to make coding decisions; (b) trying out various search strategies in eHRAF's advanced search function to evaluate what combinations of subject categories and/or keywords best find paragraphs with appropriate information; (c) RAs (ideally two) each making an independent pass through the sample societies, rating only one variable or a narrow domain at a time for a predefined time and place focus; and (d) reliability being assessed and resolutions made.

In Pulotu, the RAs were given a period of initial training followed by a team approach involving dialogue and ongoing feedback with the more experienced team members. The relatively small number of ethnographic sources involved meant that these senior team members became very familiar with the available literature on the societies being coded. The codings provided by RAs were linked to reference sources and page numbers on the Pulotu website. The NHS Ethnography similarly used teams of annotators and coders who familiarised themselves with portions of ethnographies, identified through keyword searches, contained in eHRAF.

Approaches such as these, which take as their source material a circumscribed and relatively small corpus of ethnographies, are not directly scalable when dealing with culture-level generalisations about very large-scale societies, where the number of materials to be mastered would be overwhelming. This points to one of the limitations of the RA approach.

Seshat provides another model for RA-sourced data that encompasses both small-scale and large-scale societies. The codings provided by RAs are intended to be accompanied by justifications, including references and page numbers, in a public website. This allows an evaluation of the quality of sources used and also facilitates replication efforts: other RAs can review the same materials and compare their codings to assess inter-coder reliability. An effort is made to contact experts to review the codings and suggest corrections, and outside experts viewing the codings on the Seshat site can also contact the team with suggested corrections. In this regard, the Seshat coding strategy resembles that of Pulotu or the NHS, although when dealing with large-scale societies a greater degree of expert guidance and feedback is required to choose appropriate secondary sources, interpret them correctly, etc.

**Figure 2.**
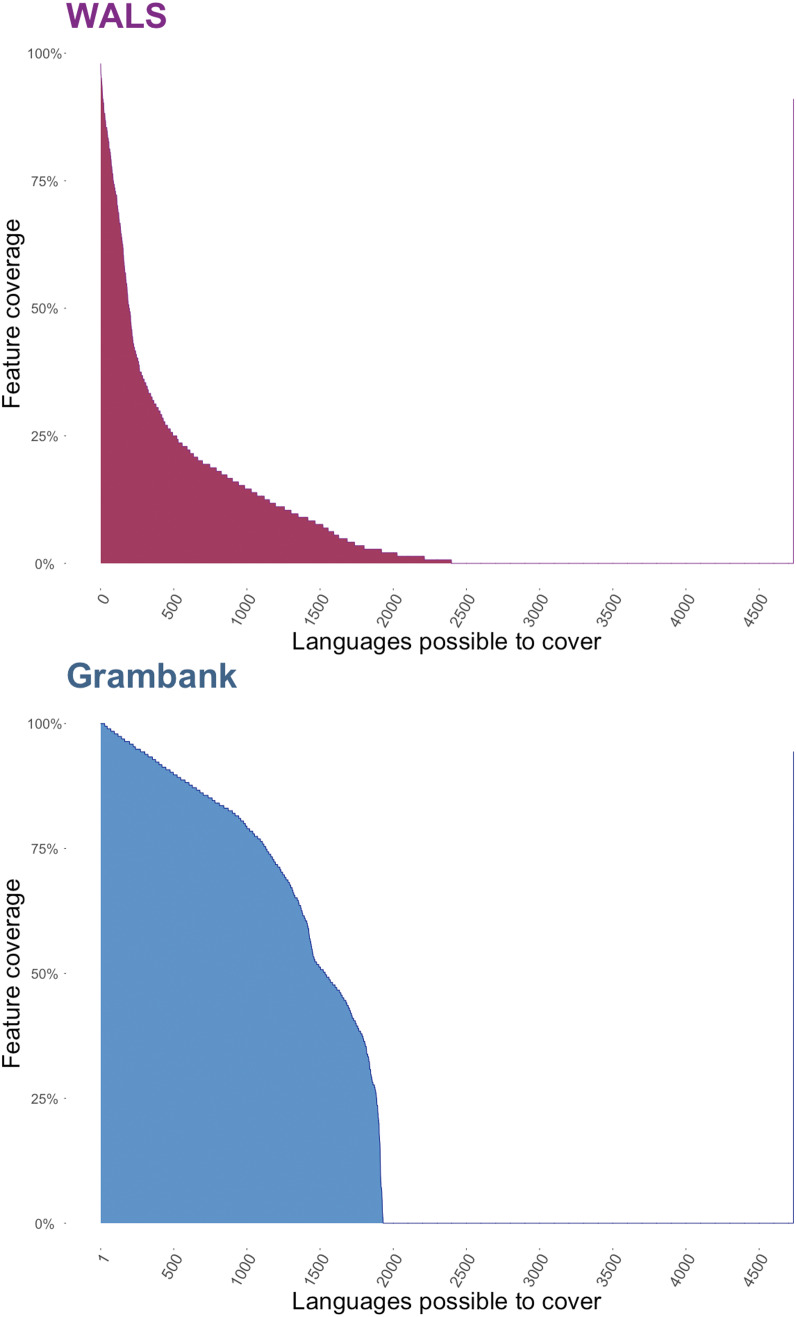
A comparison of the feature coverage in WALS, which employed an expert coding model, and Grambank, which employed a hybrid RA + Expert coding model. WALS coded 138 ‘core’ features (193 total), covered 2,679 languoids (2,466 unique languages) and had a mean feature coverage of only 18% per language. In contrast, Grambank currently codes 195 features for 1,478 languoids (1,456 unique languages) and has a mean feature coverage of 69% per language.

The linguistic database project Grambank employs a hybrid coding model. Coders are trained to fill in the questionnaire by local supervisors who were involved in the design and ongoing curation of Grambank features. Training consists of coding a previously coded language, detailed supervisor-led discussion of each questionnaire feature, introduction to the project's documentation and discussion forum and examination of previous discussions and complicated coding decisions. A key feature of the Grambank coding process is that discussions are led by one of the feature experts, known internally to the project as ‘patrons’. In cases where there is doubt or disagreement about specific coding decisions, the patron makes the final judgment. A full record of these discussions and documentation of each feature can be found in the Grambank GitHub repository. This hybrid coding strategy ensures consistency across coders and good coverage of features (see Figure 2), and provides rich documentation of the decisions required to convert the complexity of a grammatical description into a digital database in a transparent and reproducible manner.

An alternative to the RA approach is to induce experts in a relevant field – historians, archaeologists, religious studies scholars, etc. – to perform the coding themselves. The most obvious advantage of this approach is that experts who have spent much of their career studying the relevant culture, and are deeply immersed in both primary and secondary sources, are arguably best qualified to make challenging coding decisions. This is particularly important when dealing with complex, large-scale cultures. At least in principle, the expert strategy also avoids data bottlenecks, since participation is generally voluntary or minimally financially compensated. The greatest challenge is that it is, in fact, difficult to convince humanities scholars to lend their expertise to database projects. Such participation is not normative in humanities fields, which reduces incentives; in addition, many, if not most, humanities scholars are suspicious or openly hostile towards attempts to code the historical record and subject it to mathematical analysis (Slingerland & Sullivan, 2017). The difficulty of getting expert participation means that data-gathering progress in expert-based projects is generally slow and difficult to predict, and often results in undesirably skewed datasets. For instance, as one can see in Figure 2, the expert-centred WALS project has a mean linguistic feature coverage of only 18% per language, because the individual experts who have contributed have dealt only with the specific features that interest them personally. On the other hand, the Grambanks hybrid RA + expert model, where more complete coverage of features is driven by the RAs, has resulted in a current mean coverage of 69%. Idiosyncratic theoretical commitments may also cause certain experts to interpret data in a manner that is less accurate than that of a well-trained RA, although this danger can be mitigated by having multiple, overlapping codings from a representative spectrum of a given field.

The DRH is one large comparative database that is designed to rely primarily on expert-sourced data. Experts are actively recruited and vetted by an editorial team, who also approve published entries. Ideally, a large proportion of experts worldwide who study the history or archaeology of religion will eventually contribute one or more entries, resulting in massive amounts of overlapping data that will allow researchers to see areas of consensus and disagreement and to weight analyses accordingly. DRH coverage to date, however, reflects the weaknesses of the expert-centred approach. Although in its second full year of active data gathering, the DRH has comparatively little data. The data it does have is also very unevenly distributed across the globe and through time, because of the reliance on volunteer experts (current coverage can be viewed at https://religiondatabase.org/browse/).

Between the RA- and expert-based strategies various hybrid models are possible, which can help to correct the excesses of relying too heavily upon one or the other. One such model might be computer-assisted coding, where initial text mining is followed by RA and expert vetting. This is the approach adopted by the Natural History of Song Project. This strategy is also currently being explored by the Seshat team (Turchin et al., 2015). Coding decisions made by RAs can also be vetted by experts, which usually reduces the demands on the expert and therefore increases the likelihood of participation. This is Seshat's primary strategy, although the degree of expert guidance and vetting that has actually been obtained is difficult to assess (Slingerland et al., 2020). In addition to expert-created entries, the DRH has ‘Secondary Source’ entries, prepared by RAs based on a literature review, as well as ‘Expert Source’ entries, where an advanced PhD student in a relevant field completes a coding set using the published works of a given expert, and the coding decisions and comments are then reviewed, corrected and only published once approved by the expert. More of a focus on these entry types might help the DRH to boost its data quantity and improve its evenness of coverage.

## Transparency: evaluating evidence, incorporating uncertainty and facilitating reproducibility

It is more the rule than the exception that codings of cultural data are characterised by high degrees of both uncertainty and disagreement among experts, in which cases the ability to capture multiple opinions (DRH, Seshat) and interobserver reliability coding (Grambank), and enable expert commentary and/or editing (DRH, Seshat, Grambank) is desirable.

It is desirable to reflect the degree of uncertainty of a given code, whether this uncertainty stems from insufficient evidence or lack of consensus among experts. Coders themselves can indicate their degree of confidence in their coding decisions using a sliding scale or graded options (‘Yes’, ‘Inferred Yes’, ‘No’, ‘No based on lack of evidence’, etc.), but this itself reflects an individual judgment call. Alternately, specific codes can be developed to assess data quality for each variable coded (Ember & Ember, 2009), such as whether there is extensive documentation or discussion of a specific topic, whether the topic is based on scattered or anecdotal evidence, or whether the information is contradictory. The additional cognitive load involved in confidence decisions makes such measures difficult or impossible to implement with expert volunteers.

An alternative method for assessing uncertainty, adopted by the DRH, is to poll a large number of experts or RAs coding identical or overlapping entries and to allow vetted experts or RAs to provide confirmations or alternative answers to previously coded entries. This provides a measure of the degree of consensus on a given topic, and has the added bonus of highlighting promising areas of research for young scholars. However, a drawback of this method is that the different people polled might not understand the specific coding parameters, such as the time and place foci for the original coding.

The processes by which qualitative cultural information, such as historical and ethnographic evidence, are converted to quantitative data vary widely depending upon the research team, the type of data and the purpose of coding. However, the protocol for this conversion is sometimes poorly documented and difficult to replicate. Moreover, cultural data are rich and dense in some regions or time periods while being sparse in others. The social science replication crisis and open science movement have revealed the dangers of researcher degrees of freedom (Munafò et al., 2017). The potential crisis is more severe in this qualitative to quantitative conversion when researcher decisions are undocumented and researcher degrees of freedom unaccountably larger.

Transparency is crucial. While there can be reasonable disagreement over appropriate data gathering strategies for a given project, what is not debatable is that research teams need to be fully transparent about the methods actually employed to produce the data used in published analyses. It is very difficult to evaluate the reliability of any comparative cultural analysis in the absence of full documentation concerning data sourcing, rationales for coding decisions, references consulted and the presence or absence of expert vetting. Practically speaking, journal editors and referees should require full data deposition and clear, public attribution of codings and presence or absence of expert vetting. This allows independent assessment of the reliability of the underlying data, makes it possible for analysts to employ appropriate statistical controls for data uncertainties and biases and enables independent replications.

Lack of transparency has led to replication crises in other fields (Christensen & Miguel, 2018; Collaboration, 2012; Sullivan et al., 2017) and risks undermining published analyses in this burgeoning field (e.g. Slingerland et al., 2020; Whitehouse et al., 2020). Peer review of studies employing coded historical data typically focuses solely on the theoretical arguments and statistical methods, with the coding itself simply accepted as if it were a report of raw data. This overlooks the challenges inherent to converting qualitative to quantitative when it comes to cultural data. The coding process itself should always be subject to peer review by relevant, qualified experts – historians, religious studies scholars and/or archaeologists.

## Winning the buy-in of cultural experts

Any big data approach to cross-cultural research is fundamentally dependent on the integrity of coding decisions. The majority of historians, archaeologists and religious studies experts, however, have no intellectual interest in cultural evolutionary analyses, and no professional incentives to participate in such projects. This means that, in order to acquire expert assistance on a significant scale, research teams need to make their platform useful for humanities scholars on their own terms, as tools for better or more easily performing traditional forms of research. This requires prioritising the building of features that scholars in the field want, which may differ from the priorities of the research team itself.

**Figure 3.**
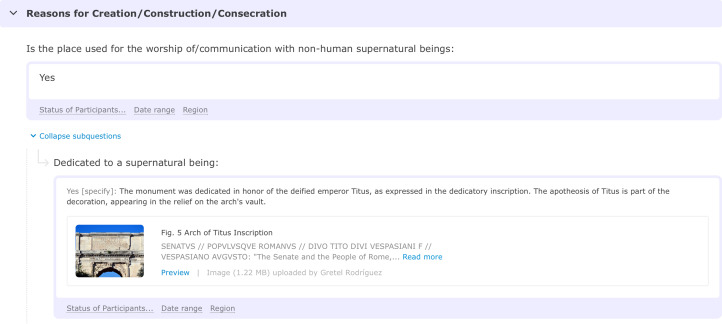
Rich data added to DRH Entry, ‘Arch of Titus’ (Places Poll), by Greta Rodrígez.

The Pulotu database, for example, provides a web interface to navigate and display the data, including maps, culture-by-culture character data and links to external sources. In the case of the DRH, it was realised early on that, for the vast majority of traditional scholars, the only way to make the DRH intrinsically motivating was to characterise it as a necessary solution to a new problem: the challenge of making and evaluating scholarly generalisations in an era of information overload (Slingerland & Sullivan, 2017). The DRH has also prioritised functions such as visualisation or the ability to add rich qualitative content (Figure 3) that appeal specifically to humanities scholars over functions that allow data to be analysed, which appeal more to scientists. It has also incentivised contribution by providing DOIs for entries and working towards becoming listed on a major scholarly citation index, which is especially crucial for promotion and tenure for humanities scholars working in Asia and Europe.

Additionally, a basic, but easy-to-overlook, requirement for a platform to become widely adopted is its basic user-friendliness and reliability. RAs can be paid to put up with clunky websites or simple spreadsheets, but winning expert participation requires a slick, intuitive and fully reliable interface. Experts annoyed by bugs or crashes will rarely come back again for a second try. Given the variety of possible browsers and devices, achieving this is surprisingly difficult and expensive. Research teams need to build in appropriate funding, make use of already-existing platforms or keep things simple – by, for instance, simply displaying data with simple text and minimising interactive ability (see, e.g. the interface for the ‘Our World in Data’ site at https://ourworldindata.org/).

In the longer term, the study of cultural evolutionary dynamics on a large scale has the best chance of success if it can forge deep and substantive links with humanities scholars and encourage genuine changes in normative scholarly practice in the humanities.

For instance, Charles Muller, the creator of the Digital Dictionary of Buddhism (http://www.buddhism-dict.net/dicts-intro.html), struggled for over a decade to induce scholars in the field to begin contributing data. Once he reached a critical mass of contributors, however, contributing to the forum became a normative practice in Buddhist studies, and it is now a comprehensive, standard and constantly growing reference source. For an expert-sourced platform to succeed, contributing to it must become similarly normative for scholars of religion, archaeologists, linguists, and historians.

Evolutionary biology provides one aspirational model that demonstrates how databases can improve standards and foster open science in a field. Genbank (https://www.ncbi.nlm.nih.gov/genbank/) is an annotated collection of all publicly available DNA sequence data, supported and maintained by the National Centre for Biotechnology Information (part of the National Institutes of Health in the United States). The database has become a universal standard for the submission of sequence data to Genbank and reporting of accession numbers is now a requirement of most journals in the field. This has made more than 200 million sequences freely accessible to anyone. A standard annotation format makes accessing the data straightforward and has spawned numerous third-party tools for searching and manipulating Genbank's treasure trove of data.

## Sustainability and extensibility

Most existing cross-cultural databases cannot be linked to others, and lack basic features that would make their data more broadly usable. These include APIs, which allow databases to dynamically access and mirror information from other databases, or more simply common file export formats, such as csv files. Moreover, these databases are often built by non-experts, which can sometimes lead to poor practices in backup procedures, data preservation and choice of technologies. In order to establish a permanent digital ecosystem for comparative cultural history, standardisation and interoperability are a necessity and best practices a safer path. Consideration of data sustainability is crucial to ensure that these data gathered at great cost and effort will remain accessible over the long term.

One emerging problem in the field is a proliferation of bespoke databases, many of which are not publicly accessible, and very few of which allow easy downloading or export of their data. Huge quantities of grant money are expended to create one-off databases that then sit, unused, on researchers’ hard-drives or difficult-to-use websites, and that eventually become completely inaccessible because of changes in technology (Ember et al., 2013). Centralised but open and expandable database platforms like Grambank and the DRH are one solution to this problem. In any case, if databases are made publicly available and follow basic interoperability standards, the data they contain can continue to be a resource for the research community and be incorporated into larger platforms through APIs. Such standards have not coalesced, but a basic starting point would be to, whenever possible, employ open source technology with open licences, built on easily maintained technology stacks.

Four goals for developing good software are efficiency, dependability, maintainability, and usability (Sommerville, 2016). There are some basic principles that can help researchers to meet these goals. Academics are often forced to write their own software owing to budget constraints, but where possible, working in collaboration with commercial or academic software engineers will lead to better code and better guidance for important decisions, such as technology choices. Professionals are also more likely to develop efficient (e.g. fast and memory-frugal) and dependable (e.g. secure, reliable and bug-free) software. The software will be easier to maintain, and therefore more future-proof, if popular technologies, programming languages and data formats are used. Using more popular technologies also means that there is a larger pool of engineers who can work on the system and a larger community and support corpus if things go wrong. There should be a clear plan for how to replace or migrate away from any particular technology.

For example, if your database relies heavily on Google's Data Studio (datastudio.google.com), there needs to be a plan for what happens if Data Studio joins the Google graveyard (killedbygoogle.com). Migration is also easier if the software is designed in a modular, encapsulated manner, such as by using object-oriented programming (McConnell, 2004) or model-view-controller design (MVC; Reenskaug & Coplien, 2009). Both paradigms try to separate the components of a system as much as possible, so that any part can be rewritten or replaced without affecting other parts of the system. For example, under a MVC database design, the actual database is abstracted as a ‘model’ and is separated from the user interface by the ‘view’, which shows the user what they need to see, and the ‘controller’, which allows the user to indirectly change the model. Ultimately, this means that the user interface can be completely rewritten without affecting the underlying database. Similarly, choosing a popular, well-established, powerful and open-source backend database technology, such as postgreSQL (postgresql.org), helps guard against the risk of a particular company's collapse or decision to stop supporting a chosen technology. The advantage of a technology such as postgreSQL is that, were it to disappear overnight, it could be replaced with another database technology while retaining all data and with minimal effects on other parts of the system (assuming an MVC design was used). This same paradigm can be applied to hardware design by automating server management, where the server is set up and controlled by code (e.g. ansible.com) and by containerising, where the server environment is virtual rather than physical (e.g. docker.com). This also allows all code to be easily version controlled (e.g. git-scm.com), reviewed, tested and automatically deployed (continuous integration (Duvall et al., 2007).

Poor usability – the system not behaving as its intended audience expects – emerges not only because the developers do not understand the users, but also because developers and academics often do not understand each other. Interdisciplinary collaboration is a challenge even between closely related fields. Between researchers and developers there is often an even larger gap between understanding what is easily technologically possible and what is actually required for the research. The use of incremental, iterative, software development processes, whereby software is built in small pieces with regular feedback from users (e.g. Agile; Rubin, 2012), allows academic experts to offer feedback early and regularly to ensure that what is built is what is needed and intended.

These practices are commonplace throughout the commercial world, including in large database projects. For example, these practices allow MediaWiki, the technology behind Wikipedia, to be installed using a variety of database technologies (e.g. MySQL, PostgreSQL, SQLite; https://en.wikipedia.org/wiki/MediaWiki#Database). Most of these practices are also employed by the Open Science Framework (osf.io), which is built on the Django MVC framework (github.com/CenterForOpenScience/osf.io).

Finally, an enormous amount of work and effort is required to create highly user-friendly, flexible, general-purpose platforms for entering, sharing, storing, analysing and visualising historical data, but the resulting product can become a general resource for the broader research community. The DRH, for instance, began with Religious Group as its analytical unit, but new polls allow experts to choose other units, and a plethora of future polls, each based on a different unit of analysis and expanding to non-religious data, are possible. Independent research projects are already using the DRH as a platform to host their own polls and visualise and analyse their data, which can be viewed separately or in conjunction with other DRH data. Common tagging systems (e.g. religious group, geographic region), grounding in space and time, as well as background links between related questions, allow all of the data, regardless of poll type, to be easily integrated. Given the emergence of such flexible, multiple-use platforms, research teams should make more of an effort to plug into existing projects, rather than creating their own. Granting agencies should encourage this practice in order to safeguard the continued usefulness of the data gathered as a result of their funding.

## Specific recommendations

We conclude by offering specific recommendations for maximising the use of existing cultural databases and designing future projects or add-ons to existing projects. This list of desiderata should also help to establish some fundamental best practices for performing this sort of research, allowing more rigorous peer-review of studies and grant proposals, and ensuring the quality of the resulting data.

*1. Think long and hard about the choice of appropriate units and the sampling strategy.* The cultural units should be carefully chosen to ensure that they are directly comparable and have a specific time and place focus. The traits need to selected to ensure that they can be reliably coded and are described at the right level of granularity to answer the key questions driving the study. Similarly the sampling strategy should be designed to answer these key questions rather than simply reflecting the data was easiest to collect.

*2. Link all data to GIS maps and standardised date formats.* Whatever unit of analysis is chosen to organise the collection and coding of data, all data should be grounded in space and time by being linked to a GIS map and date or date range. This allows outside research groups to access the data in searches and coordinate with other variables.

*3. Incorporate uncertainty.* In order to capture uncertainty, whether stemming from sparseness of sources or expert disagreement, codes for any given variable should allow for degrees of uncertainty. This can take the form of value ranges (e.g. population estimates), coder confidence ratings or overlapping values from different coders. The architecture of the database should ideally not force a single value upon variables.

*4. Make all coding, coding justifications and coding personnel involvement public.* For a comparative science of culture to succeed it should also become a basic principle of scholarly ethics to only publish papers where the data – the codes, coding justifications and identity of the coder – has been made clearly and publicly available. In cases when a coding was performed by an RA, the qualifications of the RA should be made clear. If experts were involved in reviewing or vetting codes, this involvement must be clearly explained and linked to individual data points. Specific references should be cited identifying the page number of the source. Journals and reviewers should refuse to consider any manuscript that does not follow this practice.

*5. Encourage involvement of experts.* The creators of large databases, who are often cultural evolutionary theorists, should perform outreach with relevant expert communities, encouraging their involvement in choice of units of analysis, design of coding questions and rubrics, and ideally in the coding itself or the vetting of coding performed by RAs. This buy-in will be facilitated by making the database site itself user-friendly and easy to navigate, and by building in features desired by the experts themselves.

*6. Involve humanities scholars in peer review.* Coding historical cultural data is challenging and allows many degrees of freedom. The coding process itself must be subject to peer review, which requires that journal editors reach out beyond their typical referee networks to bring in historians, religious studies scholars and/or archaeologists. Ensuring the integrity of the underlying data in a submitted paper should be the first priority; only then does it make sense to evaluate the higher-level analyses and theoretical interpretations.

*7. Use open-source technologies and have a maintenance plan.* When possible, leverage existing platforms that make themselves available to outside research groups. If building your own database, use open-source technologies and have a concrete plan for database maintenance and software updating. Future-proof your project to avoid the loss of data and waste of scholarly resources.

## Conclusion

This is an exciting time to be doing large-scale, cross-cultural analyses. The affordances of digital technologies make entirely new analyses possible. However, these novel research possibilities combine promise and perils. To avoid wasted effort, fragmentation of resources and the production of data undermined by concerns about quality or reproducibility, it is imperative that a basic set of best practices be established now. Since following such practices is neither convenient nor immediately rewarding, progress requires that journals, reviewers and funding agencies take responsibility for enforcing their adoption.

Done properly, the comparative science of culture can produce findings that will be taken seriously by scholars in the humanities, essential partners in the development of the field, and that can also serve as a solid foundation for future scientific work.
